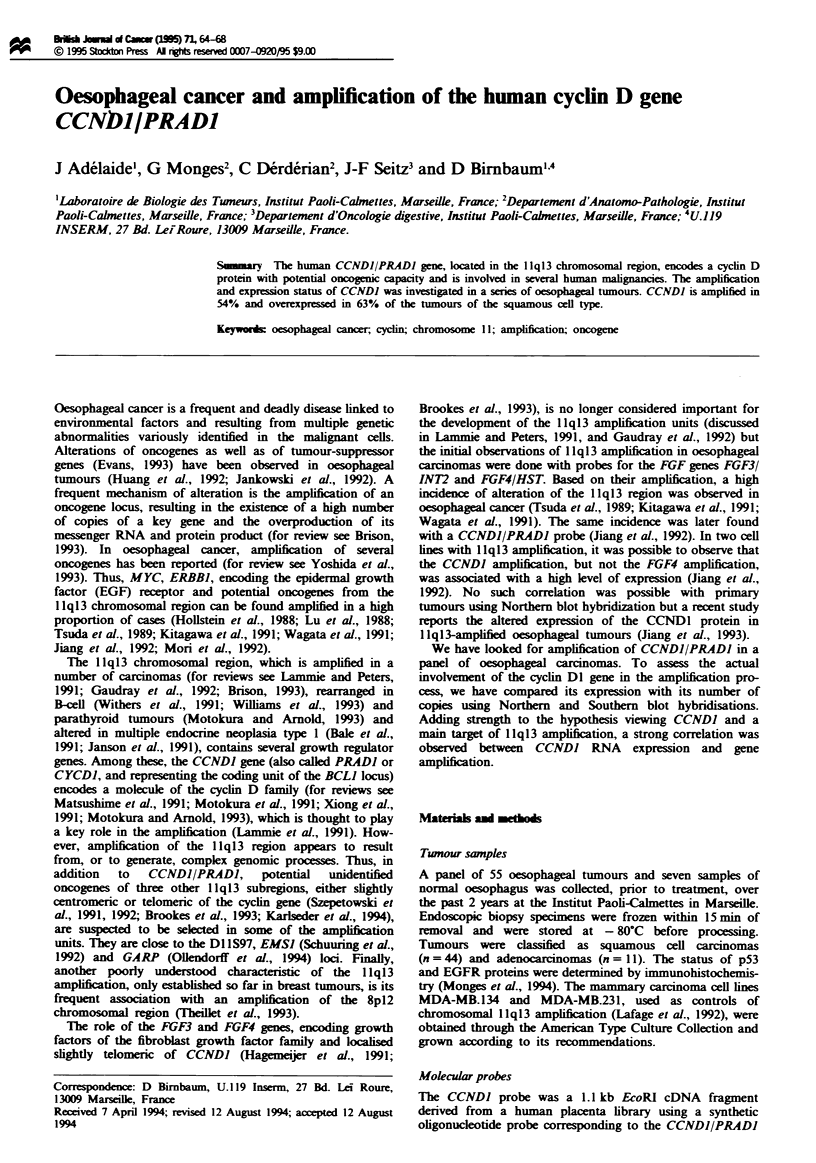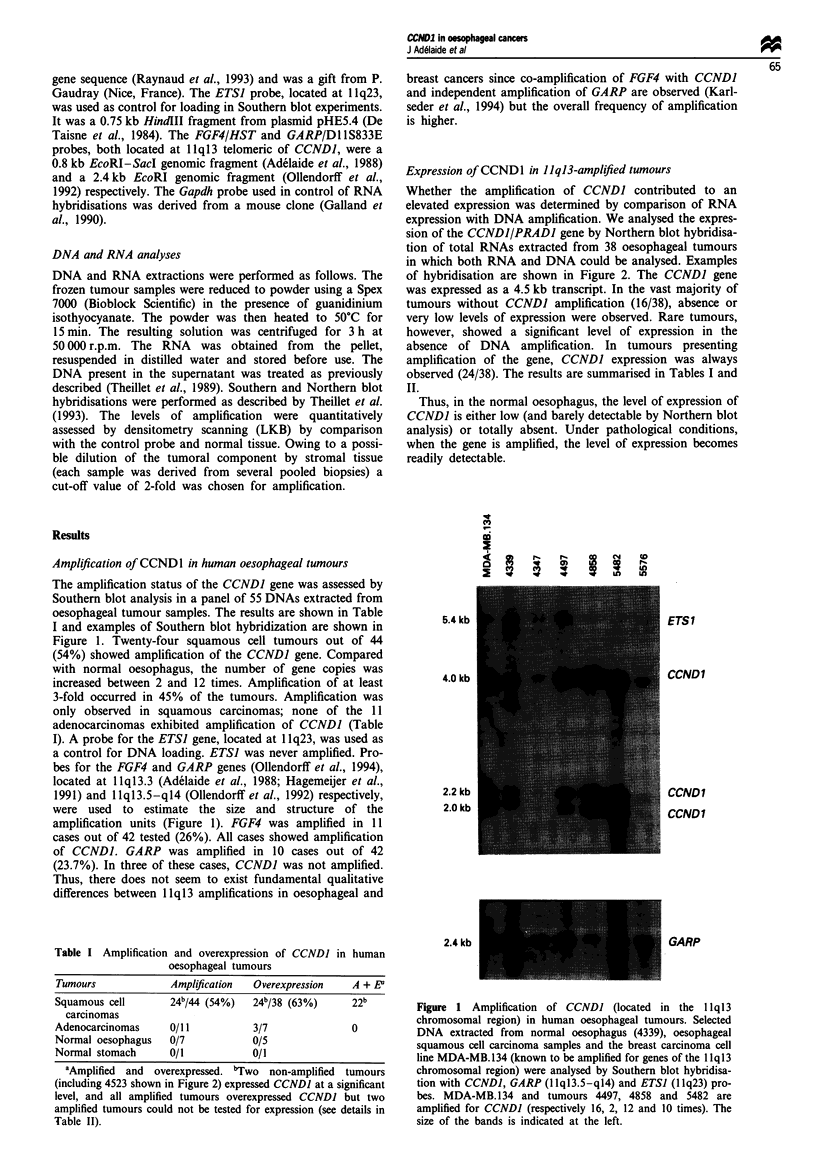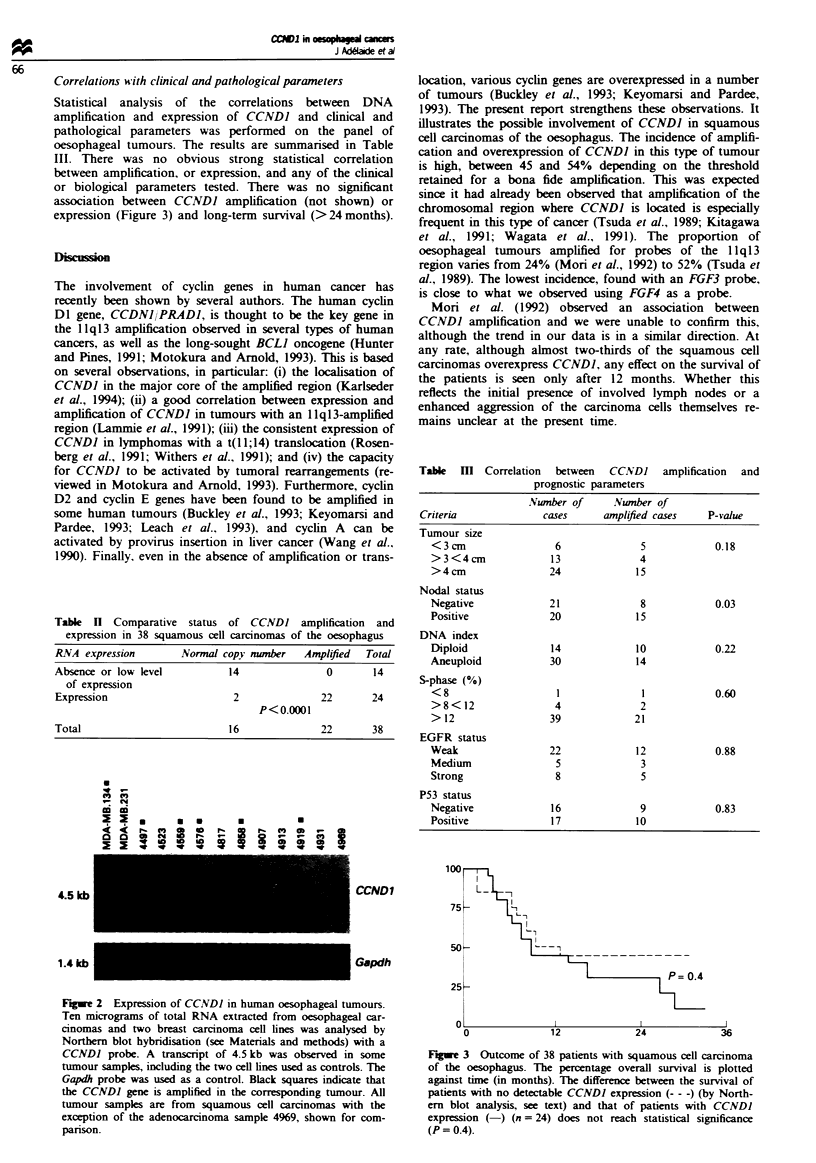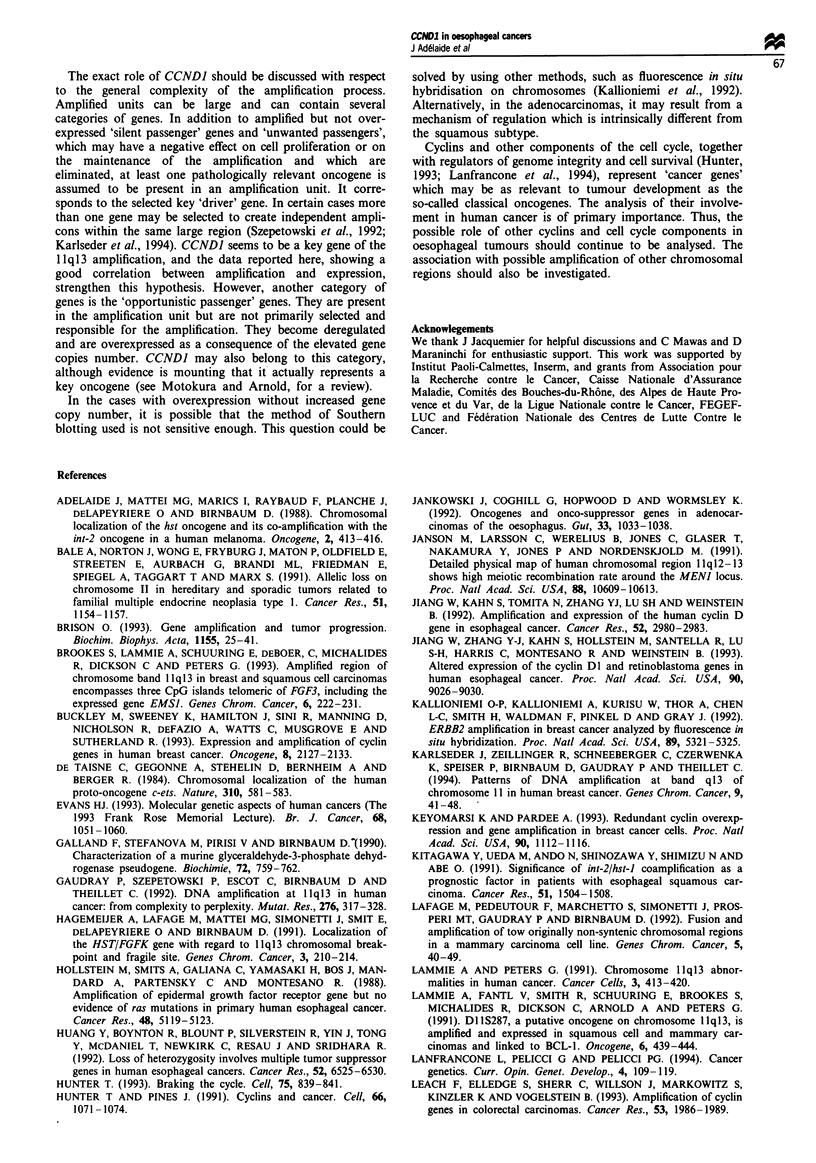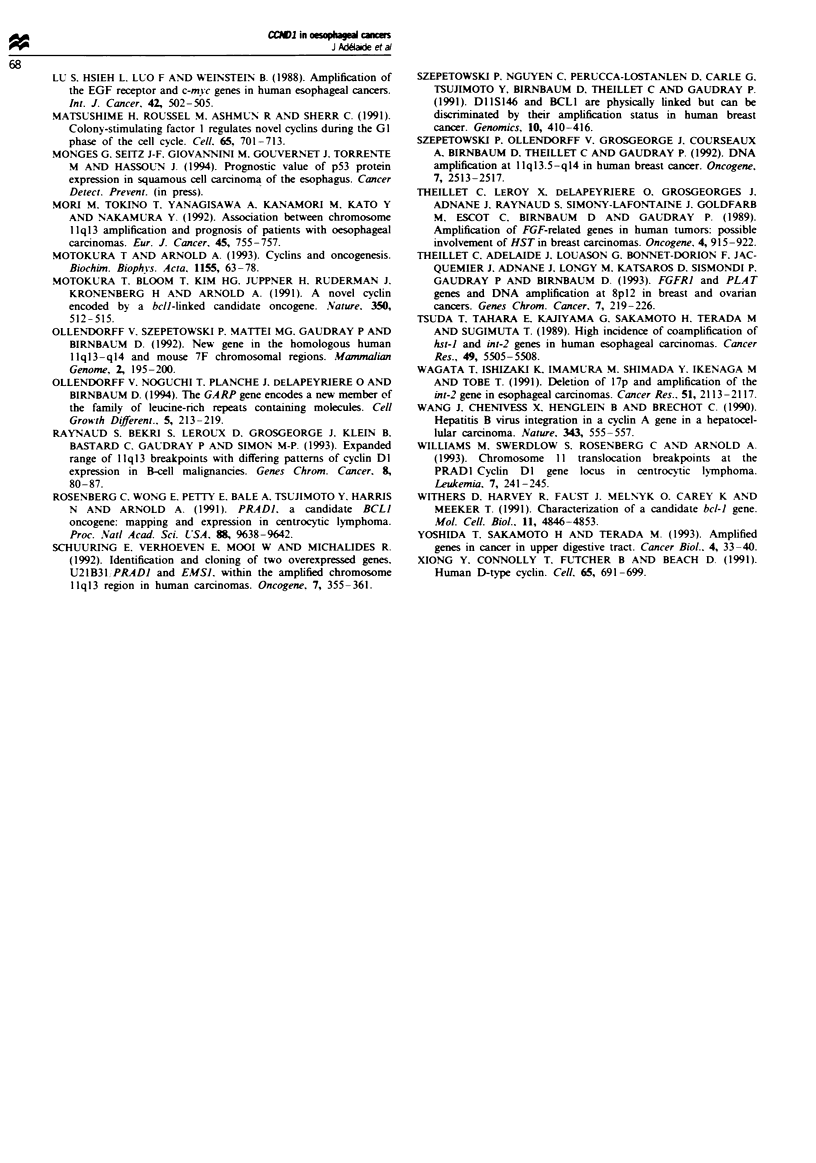# Oesophageal cancer and amplification of the human cyclin D gene CCND1/PRAD1.

**DOI:** 10.1038/bjc.1995.13

**Published:** 1995-01

**Authors:** J. Adélaide, G. Monges, C. Dérdérian, J. F. Seitz, D. Birnbaum

**Affiliations:** Laboratoire de Biologie des Tumeurs, Institut Paoli-Calmettes, Marseille, France.

## Abstract

**Images:**


© 1995 Stockton Press All rights reserved 0007–0920/95 $9.00

### Oesophageal cancer and amplification of the human cyclin D gene *CCND1/PRAD1*

J Adélaide[1], G Monges[2], C Dérdérian[2], J-F Seitz[3] and D Birnbaum[1,4]

[1]*Laboratoire de Biologie des Tumeurs, Institut Paoli-Calmettes, Marseille, France;* [2]*Departement d'Anatomo-Pathologie, Institut Paoli-Calmettes, Marseille, France;* [3]*Departement d'Oncologie digestive, Institut Paoli-Calmettes, Marseille, France;* [4]*U.119 INSERM, 27 Bd. Leï Roure, 13009 Marseille, France.*

**Summary** The human *CCND1/PRAD1* gene, located in the 11q13 chromosomal region, encodes a cyclin D protein with potential oncogenic capacity and is involved in several human malignancies. The amplification and expression status of *CCND1* was investigated in a series of oesophageal tumours. *CCND1* is amplified in 54% and overexpressed in 63% of the tumours of the squamous cell type.

**Keywords:** oesophageal cancer; cyclin; chromosome 11; amplification; oncogene

Oesophageal cancer is a frequent and deadly disease linked to environmental factors and resulting from multiple genetic abnormalities variously identified in the malignant cells. Alterations of oncogenes as well as of tumour-suppressor genes (Evans, 1993) have been observed in oesophageal tumours (Huang *et al*., 1992; Jankowski *et al*., 1992). A frequent mechanism of alteration is the amplification of an oncogene locus, resulting in the existence of a high number of copies of a key gene and the overproduction of its messenger RNA and protein product (for review see Brison, 1993). In oesophageal cancer, amplification of several oncogenes has been reported (for review see Yoshida *et al*., 1993). Thus, *MYC*, *ERBB1*, encoding the epidermal growth factor (EGF) receptor and potential oncogenes from the 11q13 chromosomal region can be found amplified in a high proportion of cases (Hollstein *et al*., 1988; Lu *et al*., 1988; Tsuda *et al*., 1989; Kitagawa *et al*., 1991; Wagata *et al*., 1991; Jiang *et al*., 1992; Mori *et al*., 1992).

The 11q13 chromosomal region, which is amplified in a number of carcinomas (for reviews see Lammie and Peters, 1991; Gaudray *et al*., 1992; Brison, 1993), rearranged in B-cell (Withers *et al*., 1991; Williams *et al*., 1993) and parathyroid tumours (Motokura and Arnold, 1993) and altered in multiple endocrine neoplasia type 1 (Bale *et al*., 1991; Janson *et al*., 1991), contains several growth regulator genes. Among these, the *CCND1* gene (also called *PRAD1* or *CYCD1*, and representing the coding unit of the *BCL1* locus) encodes a molecule of the cyclin D family (for reviews see Matsushime *et al*., 1991; Motokura *et al*., 1991; Xiong *et al*., 1991; Motokura and Arnold, 1993), which is thought to play a key role in the amplification (Lammie *et al*., 1991). However, amplification of the 11q13 region appears to result from, or to generate, complex genomic processes. Thus, in addition to *CCND1/PRAD1*, potential unidentified oncogenes of three other 11q13 subregions, either slightly centromeric or telomeric of the cyclin gene (Szepetowski *et al*., 1991, 1992; Brookes *et al*., 1993; Karlseder *et al*., 1994), are suspected to be selected in some of the amplification units. They are close to the D11S97, *EMS1* (Schuuring *et al*., 1992) and *GARP* (Ollendorff *et al*., 1994) loci. Finally, another poorly understood characteristic of the 11q13 amplification, only established so far in breast tumours, is its frequent association with an amplification of the 8p12 chromosomal region (Theillet *et al*., 1993).

The role of the *FGF3* and *FGF4* genes, encoding growth factors of the fibroblast growth factor family and localised slightly telomeric of *CCND1* (Hagemeijer *et al*., 1991; Brookes *et al*., 1993), is no longer considered important for the development of the 11q13 amplification units (discussed in Lammie and Peters, 1991, and Gaudray *et al*., 1992) but the initial observations of 11q13 amplification in oesophageal carcinomas were done with probes for the *FGF* genes *FGF3/INT2* and *FGF4/HST*. Based on their amplification, a high incidence of alteration of the 11q13 region was observed in oesophageal cancer (Tsuda *et al*., 1989; Kitagawa *et al*., 1991; Wagata *et al*., 1991). The same incidence was later found with a *CCND1/PRAD1* probe (Jiang *et al*., 1992). In two cell lines with 11q13 amplification, it was possible to observe that the *CCND1* amplification, but not the *FGF4* amplification, was associated with a high level of expression (Jiang *et al*., 1992). No such correlation was possible with primary tumours using Northern blot hybridization but a recent study reports the altered expression of the CCND1 protein in 11q13-amplified oesophageal tumours (Jiang *et al*., 1993).

We have looked for amplification of *CCND1/PRAD1* in a panel of oesophageal carcinomas. To assess the actual involvement of the cyclin D1 gene in the amplification process, we have compared its expression with its number of copies using Northern and Southern blot hybridisations. Adding strength to the hypothesis viewing *CCND1* and a main target of 11q13 amplification, a strong correlation was observed between *CCND1* RNA expression and gene amplification.

### Materials and methods

### *Tumour samples*

A panel of 55 oesophageal tumours and seven samples of normal oesophagus was collected, prior to treatment, over the past 2 years at the Institut Paoli-Calmettes in Marseille. Endoscopic biopsy specimens were frozen within 15 min of removal and were stored at − 80°C before processing. Tumours were classified as squamous cell carcinomas ($n = 44$) and adenocarcinomas ($n = 11$). The status of p53 and EGFR proteins were determined by immunohistochemistry (Monges *et al*., 1994). The mammary carcinoma cell lines MDA-MB.134 and MDA-MB.231, used as controls of chromosomal 11q13 amplification (Lafage *et al*., 1992), were obtained through the American Type Culture Collection and grown according to its recommendations.

### *Molecular probes*

The *CCND1* probe was a 1.1 kb *Eco*RI cDNA fragment derived from a human placenta library using a synthetic oligonucleotide probe corresponding to the *CCND1/PRAD1*

Correspondence: D Birnbaum, U.119 Inserm, 27 Bd. Leï Roure, 13009 Marseille, France
Received 7 April 1994; revised 12 August 1994; accepted 12 August 1994

gene sequence (Raynaud *et al.*, 1993) and was a gift from P. Gaudray (Nice, France). The *ETS1* probe, located at 11q23, was used as control for loading in Southern blot experiments. It was a 0.75 kb *Hind*III fragment from plasmid pHE5.4 (De Taisne *et al.*, 1984). The *FGF4/HST* and *GARP*/D11S833E probes, both located at 11q13 telomeric of *CCND1*, were a 0.8 kb *Eco*RI–*Sac*I genomic fragment (Adélaide *et al.*, 1988) and a 2.4 kb *Eco*RI genomic fragment (Ollendorff *et al.*, 1992) respectively. The *Gapdh* probe used in control of RNA hybridisations was derived from a mouse clone (Galland *et al.*, 1990).

*DNA and RNA analyses*

DNA and RNA extractions were performed as follows. The frozen tumour samples were reduced to powder using a Spex 7000 (Bioblock Scientific) in the presence of guanidinium isothyocyanate. The powder was then heated to 50°C for 15 min. The resulting solution was centrifuged for 3 h at 50 000 r.p.m. The RNA was obtained from the pellet, resuspended in distilled water and stored before use. The DNA present in the supernatant was treated as previously described (Theillet *et al.*, 1989). Southern and Northern blot hybridisations were performed as described by Theillet *et al.* (1993). The levels of amplification were quantitatively assessed by densitometry scanning (LKB) by comparison with the control probe and normal tissue. Owing to a possible dilution of the tumoral component by stromal tissue (each sample was derived from several pooled biopsies) a cut-off value of 2-fold was chosen for amplification.

### Results

*Amplification of* CCND1 *in human oesophageal tumours*

The amplification status of the *CCND1* gene was assessed by Southern blot analysis in a panel of 55 DNAs extracted from oesophageal tumour samples. The results are shown in Table I and examples of Southern blot hybridization are shown in Figure 1. Twenty-four squamous cell tumours out of 44 (54%) showed amplification of the *CCND1* gene. Compared with normal oesophagus, the number of gene copies was increased between 2 and 12 times. Amplification of at least 3-fold occurred in 45% of the tumours. Amplification was only observed in squamous carcinomas; none of the 11 adenocarcinomas exhibited amplification of *CCND1* (Table I). A probe for the *ETS1* gene, located at 11q23, was used as a control for DNA loading. *ETS1* was never amplified. Probes for the *FGF4* and *GARP* genes (Ollendorff *et al.*, 1994), located at 11q13.3 (Adélaide *et al.*, 1988; Hagemeijer *et al.*, 1991) and 11q13.5–q14 (Ollendorff *et al.*, 1992) respectively, were used to estimate the size and structure of the amplification units (Figure 1). *FGF4* was amplified in 11 cases out of 42 tested (26%). All cases showed amplification of *CCND1*. *GARP* was amplified in 10 cases out of 42 (23.7%). In three of these cases, *CCND1* was not amplified. Thus, there does not seem to exist fundamental qualitative differences between 11q13 amplifications in oesophageal and breast cancers since co-amplification of *FGF4* with *CCND1* and independent amplification of *GARP* are observed (Karlseder *et al.*, 1994) but the overall frequency of amplification is higher.

**Table I** Amplification and overexpression of *CCND1* in human oesophageal tumours

| *Tumours* | *Amplification* | *Overexpression* | *A + E*[a] |
|---|---|---|---|
| Squamous cell carcinomas | 24[b]/44 (54%) | 24[b]/38 (63%) | 22[b] |
| Adenocarcinomas | 0/11 | 3/7 | 0 |
| Normal oesophagus | 0/7 | 0/5 | |
| Normal stomach | 0/1 | 0/1 | |

[a]Amplified and overexpressed. [b]Two non-amplified tumours (including 4523 shown in Figure 2) expressed *CCND1* at a significant level, and all amplified tumours overexpressed *CCND1* but two amplified tumours could not be tested for expression (see details in Table II).

*Expression of* CCND1 *in 11q13-amplified tumours*

Whether the amplification of *CCND1* contributed to an elevated expression was determined by comparison of RNA expression with DNA amplification. We analysed the expression of the *CCND1/PRAD1* gene by Northern blot hybridisation of total RNAs extracted from 38 oesophageal tumours in which both RNA and DNA could be analysed. Examples of hybridisation are shown in Figure 2. The *CCND1* gene was expressed as a 4.5 kb transcript. In the vast majority of tumours without *CCND1* amplification (16/38), absence or very low levels of expression were observed. Rare tumours, however, showed a significant level of expression in the absence of DNA amplification. In tumours presenting amplification of the gene, *CCND1* expression was always observed (24/38). The results are summarised in Tables I and II.

Thus, in the normal oesophagus, the level of expression of *CCND1* is either low (and barely detectable by Northern blot analysis) or totally absent. Under pathological conditions, when the gene is amplified, the level of expression becomes readily detectable.

**Figure 1** Amplification of *CCND1* (located in the 11q13 chromosomal region) in human oesophageal tumours. Selected DNA extracted from normal oesophagus (4339), oesophageal squamous cell carcinoma samples and the breast carcinoma cell line MDA-MB.134 (known to be amplified for genes of the 11q13 chromosomal region) were analysed by Southern blot hybridisation with *CCND1*, *GARP* (11q13.5–q14) and *ETS1* (11q23) probes. MDA-MB.134 and tumours 4497, 4858 and 5482 are amplified for *CCND1* (respectively 16, 2, 12 and 10 times). The size of the bands is indicated at the left.

*Correlations with clinical and pathological parameters*

Statistical analysis of the correlations between DNA amplification and expression of *CCND1* and clinical and pathological parameters was performed on the panel of oesophageal tumours. The results are summarised in Table III. There was no obvious strong statistical correlation between amplification, or expression, and any of the clinical or biological parameters tested. There was no significant association between *CCND1* amplification (not shown) or expression (Figure 3) and long-term survival (>24 months).

### Discussion

The involvement of cyclin genes in human cancer has recently been shown by several authors. The human cyclin D1 gene, *CCDN1/PRAD1*, is thought to be the key gene in the 11q13 amplification observed in several types of human cancers, as well as the long-sought *BCL1* oncogene (Hunter and Pines, 1991; Motokura and Arnold, 1993). This is based on several observations, in particular: (i) the localisation of *CCND1* in the major core of the amplified region (Karlseder *et al.*, 1994); (ii) a good correlation between expression and amplification of *CCND1* in tumours with an 11q13-amplified region (Lammie *et al.*, 1991); (iii) the consistent expression of *CCND1* in lymphomas with a t(11;14) translocation (Rosenberg *et al.*, 1991; Withers *et al.*, 1991); and (iv) the capacity for *CCND1* to be activated by tumoral rearrangements (reviewed in Motokura and Arnold, 1993). Furthermore, cyclin D2 and cyclin E genes have been found to be amplified in some human tumours (Buckley *et al.*, 1993; Keyomarsi and Pardee, 1993; Leach *et al.*, 1993), and cyclin A can be activated by provirus insertion in liver cancer (Wang *et al.*, 1990). Finally, even in the absence of amplification or translocation, various cyclin genes are overexpressed in a number of tumours (Buckley *et al.*, 1993; Keyomarsi and Pardee, 1993). The present report strengthens these observations. It illustrates the possible involvement of *CCND1* in squamous cell carcinomas of the oesophagus. The incidence of amplification and overexpression of *CCND1* in this type of tumour is high, between 45 and 54% depending on the threshold retained for a bona fide amplification. This was expected since it had already been observed that amplification of the chromosomal region where *CCND1* is located is especially frequent in this type of cancer (Tsuda *et al.*, 1989; Kitagawa *et al.*, 1991; Wagata *et al.*, 1991). The proportion of oesophageal tumours amplified for probes of the 11q13 region varies from 24% (Mori *et al.*, 1992) to 52% (Tsuda *et al.*, 1989). The lowest incidence, found with an *FGF3* probe, is close to what we observed using *FGF4* as a probe.

Mori *et al.* (1992) observed an association between *CCND1* amplification and we were unable to confirm this, although the trend in our data is in a similar direction. At any rate, although almost two-thirds of the squamous cell carcinomas overexpress *CCND1*, any effect on the survival of the patients is seen only after 12 months. Whether this reflects the initial presence of involved lymph nodes or a enhanced aggression of the carcinoma cells themselves remains unclear at the present time.

**Table II** Comparative status of *CCND1* amplification and expression in 38 squamous cell carcinomas of the oesophagus

| *RNA expression* | *Normal copy number* | *Amplified* | *Total* |
|---|---|---|---|
| Absence or low level of expression | 14 | 0 | 14 |
| Expression | 2 | 22 | 24 |
| | $P<0.0001$ | | |
| Total | 16 | 22 | 38 |

**Figure 2** Expression of *CCND1* in human oesophageal tumours. Ten micrograms of total RNA extracted from oesophageal carcinomas and two breast carcinoma cell lines was analysed by Northern blot hybridisation (see Materials and methods) with a *CCND1* probe. A transcript of 4.5 kb was observed in some tumour samples, including the two cell lines used as controls. The *Gapdh* probe was used as a control. Black squares indicate that the *CCND1* gene is amplified in the corresponding tumour. All tumour samples are from squamous cell carcinomas with the exception of the adenocarcinoma sample 4969, shown for comparison.

**Table III** Correlation between *CCND1* amplification and prognostic parameters

| *Criteria* | *Number of cases* | *Number of amplified cases* | *P-value* |
|---|---|---|---|
| Tumour size | | | |
| <3 cm | 6 | 5 | 0.18 |
| >3<4 cm | 13 | 4 | |
| >4 cm | 24 | 15 | |
| Nodal status | | | |
| Negative | 21 | 8 | 0.03 |
| Positive | 20 | 15 | |
| DNA index | | | |
| Diploid | 14 | 10 | 0.22 |
| Aneuploid | 30 | 14 | |
| S-phase (%) | | | |
| <8 | 1 | 1 | 0.60 |
| >8<12 | 4 | 2 | |
| >12 | 39 | 21 | |
| EGFR status | | | |
| Weak | 22 | 12 | 0.88 |
| Medium | 5 | 3 | |
| Strong | 8 | 5 | |
| P53 status | | | |
| Negative | 16 | 9 | 0.83 |
| Positive | 17 | 10 | |

**Figure 3** Outcome of 38 patients with squamous cell carcinoma of the oesophagus. The percentage overall survival is plotted against time (in months). The difference between the survival of patients with no detectable *CCND1* expression (- - -) (by Northern blot analysis, see text) and that of patients with *CCND1* expression (—) ($n=24$) does not reach statistical significance ($P=0.4$).

The exact role of *CCND1* should be discussed with respect to the general complexity of the amplification process. Amplified units can be large and can contain several categories of genes. In addition to amplified but not over-expressed 'silent passenger' genes and 'unwanted passengers', which may have a negative effect on cell proliferation or on the maintenance of the amplification and which are eliminated, at least one pathologically relevant oncogene is assumed to be present in an amplification unit. It corresponds to the selected key 'driver' gene. In certain cases more than one gene may be selected to create independent amplicons within the same large region (Szepetowski *et al.*, 1992; Karlseder *et al.*, 1994). *CCND1* seems to be a key gene of the 11q13 amplification, and the data reported here, showing a good correlation between amplification and expression, strengthen this hypothesis. However, another category of genes is the 'opportunistic passenger' genes. They are present in the amplification unit but are not primarily selected and responsible for the amplification. They become deregulated and are overexpressed as a consequence of the elevated gene copies number. *CCND1* may also belong to this category, although evidence is mounting that it actually represents a key oncogene (see Motokura and Arnold, for a review).

In the cases with overexpression without increased gene copy number, it is possible that the method of Southern blotting used is not sensitive enough. This question could be solved by using other methods, such as fluorescence *in situ* hybridisation on chromosomes (Kallioniemi *et al.*, 1992). Alternatively, in the adenocarcinomas, it may result from a mechanism of regulation which is intrinsically different from the squamous subtype.

Cyclins and other components of the cell cycle, together with regulators of genome integrity and cell survival (Hunter, 1993; Lanfrancone *et al.*, 1994), represent 'cancer genes' which may be as relevant to tumour development as the so-called classical oncogenes. The analysis of their involvement in human cancer is of primary importance. Thus, the possible role of other cyclins and cell cycle components in oesophageal tumours should continue to be analysed. The association with possible amplification of other chromosomal regions should also be investigated.

**Acknowlegements**
We thank J Jacquemier for helpful discussions and C Mawas and D Maraninchi for enthusiastic support. This work was supported by Institut Paoli-Calmettes, Inserm, and grants from Association pour la Recherche contre le Cancer, Caisse Nationale d'Assurance Maladie, Comités des Bouches-du-Rhône, des Alpes de Haute Provence et du Var, de la Ligue Nationale contre le Cancer, FEGEF-LUC and Fédération Nationale des Centres de Lutte Contre le Cancer.

### References

ADELAIDE J, MATTEI MG, MARICS I, RAYBAUD F, PLANCHE J, DELAPEYRIERE O AND BIRNBAUM D. (1988). Chromosomal localization of the *hst* oncogene and its co-amplification with the *int-2* oncogene in a human melanoma. *Oncogene*, **2**, 413–416.

BALE A, NORTON J, WONG E, FRYBURG J, MATON P, OLDFIELD E, STREETEN E, AURBACH G, BRANDI ML, FRIEDMAN E, SPIEGEL A, TAGGART T AND MARX S. (1991). Allelic loss on chromosome II in hereditary and sporadic tumors related to familial multiple endocrine neoplasia type 1. *Cancer Res.*, **51**, 1154–1157.

BRISON O. (1993). Gene amplification and tumor progression. *Biochim. Biophys. Acta*, **1155**, 25–41.

BROOKES S, LAMMIE A, SCHUURING E, DEBOER, C, MICHALIDES R, DICKSON C AND PETERS G. (1993). Amplified region of chromosome band 11q13 in breast and squamous cell carcinomas encompasses three CpG islands telomeric of *FGF3*, including the expressed gene *EMS1*. *Genes Chrom. Cancer*, **6**, 222–231.

BUCKLEY M, SWEENEY K, HAMILTON J, SINI R, MANNING D, NICHOLSON R, DEFAZIO A, WATTS C, MUSGROVE E AND SUTHERLAND R. (1993). Expression and amplification of cyclin genes in human breast cancer. *Oncogene*, **8**, 2127–2133.

DE TAISNE C, GEGONNE A, STEHELIN D, BERNHEIM A AND BERGER R. (1984). Chromosomal localization of the human proto-oncogene *c-ets*. *Nature*, **310**, 581–583.

EVANS HJ. (1993). Molecular genetic aspects of human cancers (The 1993 Frank Rose Memorial Lecture). *Br. J. Cancer*, **68**, 1051–1060.

GALLAND F, STEFANOVA M, PIRISI V AND BIRNBAUM D. (1990). Characterization of a murine glyceraldehyde-3-phosphate dehydrogenase pseudogene. *Biochimie*, **72**, 759–762.

GAUDRAY P, SZEPETOWSKI P, ESCOT C, BIRNBAUM D AND THEILLET C. (1992). DNA amplification at 11q13 in human cancer: from complexity to perplexity. *Mutat. Res.*, **276**, 317–328.

HAGEMEIJER A, LAFAGE M, MATTEI MG, SIMONETTI J, SMIT E, DELAPEYRIERE O AND BIRNBAUM D. (1991). Localization of the *HST/FGFK* gene with regard to 11q13 chromosomal breakpoint and fragile site. *Genes Chrom. Cancer*, **3**, 210–214.

HOLLSTEIN M, SMITS A, GALIANA C, YAMASAKI H, BOS J, MANDARD A, PARTENSKY C AND MONTESANO R. (1988). Amplification of epidermal growth factor receptor gene but no evidence of *ras* mutations in primary human esophageal cancer. *Cancer Res.*, **48**, 5119–5123.

HUANG Y, BOYNTON R, BLOUNT P, SILVERSTEIN R, YIN J, TONG Y, MCDANIEL T, NEWKIRK C, RESAU J AND SRIDHARA R. (1992). Loss of heterozygosity involves multiple tumor suppressor genes in human esophageal cancers. *Cancer Res.*, **52**, 6525–6530.

HUNTER T. (1993). Braking the cycle. *Cell*, **75**, 839–841.

HUNTER T AND PINES J. (1991). Cyclins and cancer. *Cell*, **66**, 1071–1074.

JANKOWSKI J, COGHILL G, HOPWOOD D AND WORMSLEY K. (1992). Oncogenes and onco-suppressor genes in adenocarcinomas of the oesophagus. *Gut*, **33**, 1033–1038.

JANSON M, LARSSON C, WERELIUS B, JONES C, GLASER T, NAKAMURA Y, JONES P AND NORDENSKJOLD M. (1991). Detailed physical map of human chromosomal region 11q12–13 shows high meiotic recombination rate around the *MEN1* locus. *Proc. Natl Acad. Sci. USA*, **88**, 10609–10613.

JIANG W, KAHN S, TOMITA N, ZHANG YJ, LU SH AND WEINSTEIN B. (1992). Amplification and expression of the human cyclin D gene in esophageal cancer. *Cancer Res.*, **52**, 2980–2983.

JIANG W, ZHANG Y-J, KAHN S, HOLLSTEIN M, SANTELLA R, LU S-H, HARRIS C, MONTESANO R AND WEINSTEIN B. (1993). Altered expression of the cyclin D1 and retinoblastoma genes in human esophageal cancer. *Proc. Natl Acad. Sci. USA*, **90**, 9026–9030.

KALLIONIEMI O-P, KALLIONIEMI A, KURISU W, THOR A, CHEN L-C, SMITH H, WALDMAN F, PINKEL D AND GRAY J. (1992). *ERBB2* amplification in breast cancer analyzed by fluorescence *in situ* hybridization. *Proc. Natl Acad. Sci. USA*, **89**, 5321–5325.

KARLSEDER J, ZEILLINGER R, SCHNEEBERGER C, CZERWENKA K, SPEISER P, BIRNBAUM D, GAUDRAY P AND THEILLET C. (1994). Patterns of DNA amplification at band q13 of chromosome 11 in human breast cancer. *Genes Chrom. Cancer*, **9**, 41–48.

KEYOMARSI K AND PARDEE A. (1993). Redundant cyclin overexpression and gene amplification in breast cancer cells. *Proc. Natl Acad. Sci. USA*, **90**, 1112–1116.

KITAGAWA Y, UEDA M, ANDO N, SHINOZAWA Y, SHIMIZU N AND ABE O. (1991). Significance of *int-2/hst-1* coamplification as a prognostic factor in patients with esophageal squamous carcinoma. *Cancer Res.*, **51**, 1504–1508.

LAFAGE M, PEDEUTOUR F, MARCHETTO S, SIMONETTI J, PROSPERI MT, GAUDRAY P AND BIRNBAUM D. (1992). Fusion and amplification of tow originally non-syntenic chromosomal regions in a mammary carcinoma cell line. *Genes Chrom. Cancer*, **5**, 40–49.

LAMMIE A AND PETERS G. (1991). Chromosome 11q13 abnormalities in human cancer. *Cancer Cells*, **3**, 413–420.

LAMMIE A, FANTL V, SMITH R, SCHUURING E, BROOKES S, MICHALIDES R, DICKSON C, ARNOLD A AND PETERS G. (1991). D11S287, a putative oncogene on chromosome 11q13, is amplified and expressed in squamous cell and mammary carcinomas and linked to BCL-1. *Oncogene*, **6**, 439–444.

LANFRANCONE L, PELICCI G AND PELICCI PG. (1994). Cancer genetics. *Curr. Opin. Genet. Develop.*, **4**, 109–119.

LEACH F, ELLEDGE S, SHERR C, WILLSON J, MARKOWITZ S, KINZLER K AND VOGELSTEIN B. (1993). Amplification of cyclin genes in colorectal carcinomas. *Cancer Res.*, **53**, 1986–1989.

LU S. HSIEH L. LUO F AND WEINSTEIN B. (1988). Amplification of the EGF receptor and c-*myc* genes in human esophageal cancers. *Int. J. Cancer*, **42**, 502–505.

MATSUSHIME H. ROUSSEL M. ASHMUN R AND SHERR C. (1991). Colony-stimulating factor 1 regulates novel cyclins during the G1 phase of the cell cycle. *Cell*, **65**, 701–713.

MONGES G. SEITZ J-F. GIOVANNINI M. GOUVERNET J. TORRENTE M AND HASSOUN J. (1994). Prognostic value of p53 protein expression in squamous cell carcinoma of the esophagus. *Cancer Detect. Prevent.* (in press).

MORI M. TOKINO T. YANAGISAWA A. KANAMORI M. KATO Y AND NAKAMURA Y. (1992). Association between chromosome 11q13 amplification and prognosis of patients with oesophageal carcinomas. *Eur. J. Cancer*, **45**, 755–757.

MOTOKURA T AND ARNOLD A. (1993). Cyclins and oncogenesis. *Biochim. Biophys. Acta*, **1155**, 63–78.

MOTOKURA T. BLOOM T. KIM HG. JUPPNER H. RUDERMAN J. KRONENBERG H AND ARNOLD A. (1991). A novel cyclin encoded by a *bcl1*-linked candidate oncogene. *Nature*, **350**, 512–515.

OLLENDORFF V. SZEPETOWSKI P. MATTEI MG. GAUDRAY P AND BIRNBAUM D. (1992). New gene in the homologous human 11q13–q14 and mouse 7F chromosomal regions. *Mammalian Genome*, **2**, 195–200.

OLLENDORFF V. NOGUCHI T. PLANCHE J. DELAPEYRIERE O AND BIRNBAUM D. (1994). The *GARP* gene encodes a new member of the family of leucine-rich repeats containing molecules. *Cell Growth Different.*, **5**, 213–219.

RAYNAUD S. BEKRI S. LEROUX D. GROSGEORGE J. KLEIN B. BASTARD C. GAUDRAY P AND SIMON M-P. (1993). Expanded range of 11q13 breakpoints with differing patterns of cyclin D1 expression in B-cell malignancies. *Genes Chrom. Cancer*, **8**, 80–87.

ROSENBERG C. WONG E. PETTY E. BALE A. TSUJIMOTO Y. HARRIS N AND ARNOLD A. (1991). *PRAD1*, a candidate *BCL1* oncogene: mapping and expression in centrocytic lymphoma. *Proc. Natl Acad. Sci. USA*, **88**, 9638–9642.

SCHUURING E. VERHOEVEN E. MOOI W AND MICHALIDES R. (1992). Identification and cloning of two overexpressed genes, U21B31/*PRAD1* and *EMS1*, within the amplified chromosome 11q13 region in human carcinomas. *Oncogene*, **7**, 355–361.

SZEPETOWSKI P. NGUYEN C. PERUCCA-LOSTANLEN D. CARLE G. TSUJIMOTO Y. BIRNBAUM D. THEILLET C AND GAUDRAY P. (1991). D11S146 and BCL1 are physically linked but can be discriminated by their amplification status in human breast cancer. *Genomics*, **10**, 410–416.

SZEPETOWSKI P. OLLENDORFF V. GROSGEORGE J. COURSEAUX A. BIRNBAUM D. THEILLET C AND GAUDRAY P. (1992). DNA amplification at 11q13.5–q14 in human breast cancer. *Oncogene*, **7**, 2513–2517.

THEILLET C. LEROY X. DELAPEYRIERE O. GROSGEORGES J. ADNANE J. RAYNAUD S. SIMONY-LAFONTAINE J. GOLDFARB M. ESCOT C. BIRNBAUM D AND GAUDRAY P. (1989). Amplification of *FGF*-related genes in human tumors: possible involvement of *HST* in breast carcinomas. *Oncogene*, **4**, 915–922.

THEILLET C. ADELAIDE J. LOUASON G. BONNET-DORION F. JACQUEMIER J. ADNANE J. LONGY M. KATSAROS D. SISMONDI P. GAUDRAY P AND BIRNBAUM D. (1993). *FGFR1* and *PLAT* genes and DNA amplification at 8p12 in breast and ovarian cancers. *Genes Chrom. Cancer*, **7**, 219–226.

TSUDA T. TAHARA E. KAJIYAMA G. SAKAMOTO H. TERADA M AND SUGIMUTA T. (1989). High incidence of coamplification of *hst-1* and *int-2* genes in human esophageal carcinomas. *Cancer Res.*, **49**, 5505–5508.

WAGATA T. ISHIZAKI K. IMAMURA M. SHIMADA Y. IKENAGA M AND TOBE T. (1991). Deletion of 17p and amplification of the *int-2* gene in esophageal carcinomas. *Cancer Res.*, **51**, 2113–2117.

WANG J. CHENIVESS X. HENGLEIN B AND BRECHOT C. (1990). Hepatitis B virus integration in a cyclin A gene in a hepatocellular carcinoma. *Nature*, **343**, 555–557.

WILLIAMS M. SWERDLOW S. ROSENBERG C AND ARNOLD A. (1993). Chromosome 11 translocation breakpoints at the PRAD1/Cyclin D1 gene locus in centrocytic lymphoma. *Leukemia*, **7**, 241–245.

WITHERS D. HARVEY R. FAUST J. MELNYK O. CAREY K AND MEEKER T. (1991). Characterization of a candidate *bcl-1* gene. *Mol. Cell. Biol.*, **11**, 4846–4853.

YOSHIDA T. SAKAMOTO H AND TERADA M. (1993). Amplified genes in cancer in upper digestive tract. *Cancer Biol.*, **4**, 33–40.

XIONG Y. CONNOLLY T. FUTCHER B AND BEACH D. (1991). Human D-type cyclin. *Cell*, **65**, 691–699.